# Safety and Immunogenicity of Homologous and Heterologous Adenoviral-Vectored and mRNA COVID-19 Vaccine Regimens in Radiotherapy Patients

**DOI:** 10.3390/vaccines11071135

**Published:** 2023-06-23

**Authors:** Anussara Prayongrat, Patjaya Noppaving, Thitiporn Chobarporn, Natthinee Sudhinaraset, Nattaya Teeyapun, Nussara Pakvisal, Watsamon Jantarabenjakul, Jiratchaya Sophonphan, Chawalit Lertbutsayanukul, Yong Poovorawan

**Affiliations:** 1 Division of Radiation Oncology, Department of Radiology, Faculty of Medicine, Chulalongkorn University and King Chulalongkorn Memorial Hospital, Bangkok 10330, Thailand; anussara.p@chula.ac.th (A.P.); patjaya.n20@chula.md (P.N.); chawalit.l@chula.ac.th (C.L.); 2 Department of Surgery, Faculty of Medicine, Chulalongkorn University, Bangkok 10330, Thailand; thitiporn.cho@chula.ac.th; 3 Center of Excellence in Clinical Virology, Faculty of Medicine, Chulalongkorn University, Bangkok 10330, Thailand; dr_natthinee@hotmail.com; 4 Department of Medical Oncology, Faculty of Medicine, Chulalongkorn University and King Chulalongkorn Memorial Hospital, Bangkok 10330, Thailand; nattaya.te@chula.ac.th (N.T.); nussara.p@chula.ac.th (N.P.); 5 Center of Excellence for Paediatric Infectious Diseases and Vaccines, Department of Paediatrics, Faculty of Medicine, Bangkok 10330, Thailand; watsamon.j@chula.ac.th; 6 Thai Red Cross Emerging Infectious Diseases Clinical Center, King Chulalongkorn Memorial Hospital, Bangkok 10330, Thailand; 7 HIV-NAT, Thai Red Cross AIDS Research Centre, Bangkok 10330, Thailand; jiratchaya.w@hivnat.org

**Keywords:** SARS-CoV-2, COVID-19, vaccination, radiotherapy, immunogenicity

## Abstract

Diminished immune response after vaccination occurs in cancer patients. This observational study evaluated the immune response and safety profile after COVID-19 vaccination in radiotherapy patients. The study comprised 53 cancer patients undergoing radiotherapy and voluntarily received the COVID-19 vaccine. The two regimens were homologous ChAdOx1-S recombinant (AstraZeneca, AZ), “AZ-AZ” and heterologous “AZ-mRNA”. The seroconversion rate and anti-RBD immunoglobulin geometric mean titers (GMT) were assessed and compared with healthy controls. Adverse effects were assessed using a questionnaire. The seroconversion rate was 52.4% 1 month after the first dose with GMT 4.3 U/mL (95%CI 1.4–13). Following the second dose, the AZ-AZ group achieved 95% seroconversion rate with GMT = 188.4 U/mL (95%CI 67.1–529), which was significantly lower than the healthy cohort, GMT = 945 U/mL (95%CI 708–1261). Cancer patients in AZ-mRNA group achieved a 100% seroconversion rate with a high GMT = 1400.8 U/mL (95%CI 429.5–4566), which was significantly lower than the healthy cohort, GMT = 5169.9 U/mL (95%CI 3582.2–7461.5). Most adverse effects were mild. Our findings suggest that radiotherapy patients had fair immunogenicity after the first dose, but achieved a high seroconversion rate after the second dose with manageable adverse effects. However, their immunologic response was lower than in healthy individuals, indicating that other preventive strategies are needed.

## 1. Introduction

The outbreak of coronavirus disease (COVID-19), caused by severe acute respiratory syndrome–related coronavirus (SARS-CoV-2), has had a tremendous impact on life, society, public health systems, and economies around the world since 2019. This ongoing pandemic affects the health of people in many aspects and can lead to massive damage to the body, multiple organ failure, and death. Immunocompromised hosts, including cancer patients, are highly vulnerable to the SARS-CoV-2 infection and tend to develop a severe form of COVID-19 and higher mortality rates [1,2]. Therefore, the National Comprehensive Cancer Network (NCCN) advisory committee recommends that these patients should be prioritized for COVID-19 vaccination with either an mRNA vaccine, i.e., BNT162b2 (Pfizer, PZ; BioNTech, Mainz, Germany), mRNA-1273 (Moderna, MDN; Moderna, Cambridge, MA, USA) or JNJ-78436735 (Johnson & Johnson, JJ, New Brunswick, NJ, USA; Janssen, Beerse, Belgium) [3]. 

There are several factors that may prevent the body from producing antibodies at an effective level, resulting in an insufficient immune response to COVID-19 vaccination in cancer patients. The patient factors include old age, multiple comorbidities, and intercurrent illness and medications that affect the immune boosting. Moreover, immune dysregulation usually occurs in these patients and some cancers invade the bone marrow causing reduced blood cell production. Furthermore, cancer therapies can suppress bone marrow function for weeks to months. A systematic review of 17 studies revealed that cancer patients had a lower seroconversion rate after vaccination than healthy controls with the first dose (37% vs. 74%) and the second dose (78% vs. 100%) [4]. Several studies also described the delayed and lower immune responses after COVID-19 vaccine in solid tumor patients who were undergoing systemic therapy, including chemotherapy, targeted therapy, and immunotherapy [5,6,7,8,9,10,11]. However, most studies were performed in the USA and Europe where mRNA vaccines were predominantly administered, and only a few included a small number of radiotherapy patients [8,9]. In a subset analysis of the Cancer, COVID and Vaccination cohort, Bowes et al. reported the relatively lower immune response in 33 patients who had received thoracic radiotherapy compared with the healthy controls. However, only 20% of the patients in this study received vaccination just before or during the course of radiotherapy and the type of vaccine, mRNA (PZ or MDN) and Ad26.COV2.S (Johnson & Johnson, New Brunswick, NJ, USA) vaccine, was different from our study [12].

In Thailand, the accessibility of COVID-19 vaccination in early 2021 was limited to either a whole inactivated virus COVID-19 vaccine (Sinovac), or the adenoviral-vectored ChAdOx1-nCOV-19 vaccine (AstraZeneca, AZ; AstraZeneca, Cambridge, UK). AZ has been mainly administered in cancer patients according to the Department of Public Health policy of Thailand, starting in June 2021. Subsequently, the mRNA vaccines became available in late 2021. Therefore, participants who received one dose of ChAdOx1-nCOV-19 vaccine can chose a homologous boost with ChAdOx1-nCOV-19 vaccine or a heterologous boost with mRNA vaccine. The aim of this study was to evaluate the immune response to the vaccination against COVID-19 compared with healthy controls, as well as its safety profile in radiotherapy patients in Thailand. 

## 2. Materials and Methods

### 2.1. Study Population

This study was an observational study performed in the Division of Radiation Oncology, King Chulalongkorn Memorial Hospital, Bangkok, Thailand. The patients who met the inclusion criteria were recruited and received the study information from investigators before enrolling in the study. All participants provided written informed consent prior to enrollment. The study protocol was approved by the Institutional Review Board (number 582/64) and conducted in accordance with the principles of the Declaration of Helsinki. 

### 2.2. Radiotherapy Patients

The study population comprised patients with solid tumors who received radiation treatment and voluntarily decided to receive a COVID-19 vaccination within four weeks before or after the start of radiation. The inclusion criteria were as follows: (1) age ≥ 18 years; (2) ECOG performance status 0–2; (3) well-controlled underlying disease; (4) life expectancy ≥6 months based on the patient and disease status; and (5) diagnosed with a solid cancer or a tumor requiring radiation therapy. The exclusion criteria were as follows: (1) an immunodeficiency condition such as HIV infection or congenital immunodeficiency syndrome; (2) history of COVID-19 infection or detectable nucleocapsid protein antibody before the first dose of vaccination; (3) radiation including total body irradiation, total lymphoid irradiation, or craniospinal irradiation; (4) prior radiotherapy; or (5) pregnant women. 

### 2.3. Healthy Controls

The data collection period for healthy cohort was between March 2021 and June 2021. Only age, gender, and serology data were provided. The healthy individuals were divided by age-group (<40 years, 40–60 years, and >60 years) and gender, and used as a comparison group (historical control) [13,14]. 

### 2.4. COVID-19 Vaccines

At the time of the original study design, only the AZ vaccine was allowed in cancer patients according to the national health policy. However, the mRNA vaccines, Pfizer (PZ), Moderna (MDN) vaccine, became available in Thailand in October 2021. Thus, the policy was updated to choose AZ or mRNA as the second dose. Therefore, there were two groups of patients classified by the type of vaccines, i.e., Group 1: AZ as the first dose followed by AZ as the second dose (homologous AZ-AZ) and Group 2: AZ as the first dose followed by mRNA as the second dose (heterologous AZ-mRNA). The interval between the first and second dose was 8–10 weeks for the AZ-AZ group and 4 weeks for the AZ-mRNA group. Due to slow patient accrual, the protocol was modified to include patients at the second vaccination.

### 2.5. Laboratory Tests

The serum samples were tested for total immunoglobulins (Ig) specific to the receptor-binding domain (RBD) of the SARS-CoV-2 spike (S) protein (anti-RBD Ig) using the Roche Elecsys Anti-SARS-CoV-2 S assay (Roche Diagnostics, Basel, Switzerland). According to the manufacturer’s protocol, the detection limit was 0.4 U/mL and an antibody concentration ≥0.8 U/mL was defined as seropositivity. 

The anti-nucleocapsid protein IgG test was performed using the chemiluminescent microparticle immunoassay (CMIA) (Abbott Laboratory, Chicago, IL, USA). An anti-nucleocapsid protein IgG ≥1.4 indicated natural infection of COVID-19.

Post-hoc analysis of the surrogate virus neutralization test (sVNT) against the SARS-CoV-2 variants of concern comprising the Delta (B.1.617.2) and Omicron BA.1, BA.4& BA.5 (BA.1.1.529) strain was performed in a random subset of serum samples (12 samples for the Delta strain and 32 samples for the Omicron strain) using a cPass SAR-CoV-2 neutralizing antibody detection kit (GenScript, Piscataway, NJ, USA). The method was performed as previously described [10]. Briefly, the serum samples were diluted 1:10 with dilution buffer and incubated with the Horseradish peroxidase (HRP) conjugated recombinant SARS-CoV-2 RBD fragment at 37 °C for 30 min. 100 µL of the mixture was then added to the human ACE2 receptor (hACE2) protein pre-coated plate and incubated at 37 °C for 15 min. After washing, 100 µL TMB substrate solution was added and the plate was incubated in the dark at 20–25 °C for 15 min, followed by adding 50 µL Stop solution to quench the reaction. The sample was read at 450 nm in a microtiter plate reader. Inhibition of ≥30% was considered positive.

### 2.6. Study Endpoints

The anti-RBD Ig was examined at five time points (TP) determined as TP1 (before the first dose of vaccination), TP2 (at one month after the first dose), TP3 (before the second dose), TP4 (one month after the second dose) and TP5 (three months after the second dose). For the participants who had an abnormal increase in anti-RBD Ig before each vaccination dose, the anti-nucleocapsid IgG test was performed to detect the SARS-CoV-2 infection. The participants had a complete blood count (CBC) evaluation at TP1 and TP3. Additionally, the participants completed a questionnaire of adverse events for 7 d after vaccination. 

**Figure 1:**
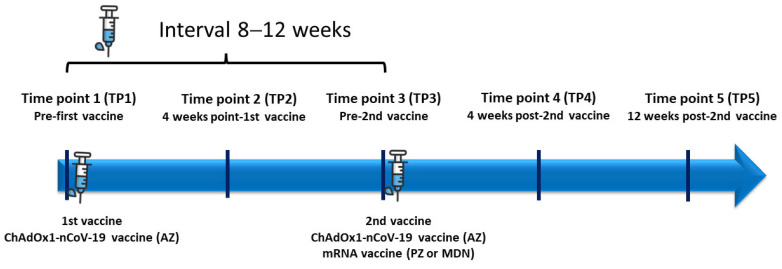
Study flow. AZ = AstraZeneca vaccine; PZ = Pfizer vaccine, Moderna vaccine.

The study flow is illustrated in Figure 1. The primary outcome of this study was to assess the seroconversion rate of anti-RBD Ig in radiotherapy patients following the first dose of COVID-19 vaccination. The secondary outcomes were to report the seroconversion rate after the booster dose and the adverse effects from COVID-19 vaccination. The results were compared with the 42 healthy cohort who received AZ-AZ vaccine from our previous studies and 16 participants and AZ-mRNA vaccine [13,14].

### 2.7. Statistical Analysis

Patients who received at least one dose of the AZ vaccine and had at least one additional immune response assessment were included in the analysis. No data imputation was performed for missing values. The anti-RBD Ig levels were reported as geometrical mean titers (GMT) with a 95% confidence interval (CI). The seroconversion rate was reported as a percentage. Comparisons were made between the radiation dose level, low-dose (<50 Gy) and high-dose (≥50 Gy), within each subgroup, which were stratified by vaccination group, was performed to determine the effect of the radiation dose on the immunogenicity of the two vaccine regimens. The non-parametric Mann-Whitney test was used to compare the anti-RBD Ig levels between subgroups. The GMT ratio (GMR) was calculated using linear regression with an outcome of log transformed antibody titer between patients vs. healthy controls, and between the low-dose vs. high-dose subgroups. Comparison of proportion for adverse effects after vaccination between two regimen and risk difference with 95%CI were calculated by two independent proportion tests. Statistical analysis was performed using Stata 15 (Statacorp LLC, College Station, TX, USA). Two-sided *p* values < 0.05 was considered significant.

## 3. Results

Fifty-three participants were included in the study between August 2021 to March 2022. The patients’ mean age was 56 years old. There were 34 females (64.2%) and 19 males (35.8%). Most of the primary tumor sites were in the chest (lung and breast) in 19 patients (35.8%), followed by the head and neck region in 17 patients (32.1%) and abdomen in 11 patients (20.7%). Most patients had stage III-IV disease. The patients’ baseline characteristics are presented in Table 1.

The median radiation dose was 45 Gy (interquartile range (IQR), 40–63) and the median number of fractions was 25 fractions (IQR, 10–30). Approximately half of the patients received a radiation dose ≥ 50 Gy. The major radiotherapy technique was intensity modulated radiotherapy (IMRT) or volumetric arc modulated radiotherapy (VMAT) technique in 31 patients (58.5%). The radiotherapy details are seen in Table 2.

A flow diagram represented data collection at each time-point (Supplementary Materials Figure S1). Before the second vaccination (TP3), there were three patients with abnormally high anti-RBD total Ig levels, thus, their blood samples were tested for anti-nucleocapsid IgG to exclude natural infection before the second vaccination. Two patients had negative results (anti-RBD total Ig = 243.1 U/mL and 225.2 U/mL). However, one patient demonstrated evidence of COVID-19 infection (anti-RBD total Ig = 524.3 U/m) and was excluded from the second analysis.

### 3.1. Immunogenicity and Serology after Vaccination

At one month following the first AZ vaccination, the seroconversion rate was 52.4% (TP2). After the second dose, the seroconversion rate in the AZ-AZ group was 95% and 89.5% at TP4 and TP5, respectively. In the AZ-mRNA group, the seroconversion rate remained 100% at both TPs. Due to different assessment protocol of laboratory testing, immunologic response at 1 month after the first dose vaccination (TP2) was not collected in healthy cohort. Therefore, the data from healthy cohort was compared at TP3, TP4, and TP5. Anticipated 100% seropositivity was seen at all available time points in the 42 healthy cohort.

**Figure 2:**
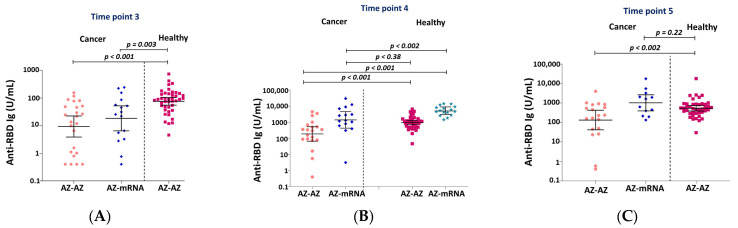
GMT and 95%CI for the anti-RBD Ig levels at time-point 3 (**A**), 4 (**B**), and 5 (**C**) comparing our patients and the healthy cohort in each vaccination regimen. Anti-RBD Ig = immunoglobulins specific to the receptor-binding domain of the SARS-CoV-2 spike protein; AZ= AstraZeneca vaccine.

The GMT of the anti-RBD total Ig levels was 4.3 U/mL (95%CI 1.4–13) in our patients at TP2. Prior to the second dose (TP3), the anti-RBD Ig level in the radiotherapy patients was significantly lower than those of healthy individuals and rose more slowly and remained significantly lower than the healthy cohort at TP4 (*p* < 0.001 in the AZ-AZ and 0.002 in AZ-mRNA regimens) and TP5 (*p* = 0.002 in the AZ-AZ and no comparison in the AZ-mRNA regimens) as shown in Figure 2. For the homologous AZ-AZ regimen, the GMT was 188.4 U/mL (95%CI 67.1–529) at TP4 and 133.1 U/mL (95%CI 41.9–423) at TP5 in the cancer patients, compared with 945 U/mL (95%CI 708–1261) and 538 U/mL (95%CI 389–744) in the healthy cohort. For the heterologous AZ-mRNA regimen, only GMT at TP4 was compared due to the limited available data in the healthy cohort. The GMT was 1400.8 U/mL (95%CI 429.5–4566) at TP4 in the cancer patients vs. 5169.9 U/mL (95%CI 3582.2–7461.5) at TP4 in the healthy cohort. The antibody titers in the patients who received the heterologous AZ-mRNA vaccine were higher than healthy controls who received AZ-AZ, but not significantly different (*p* = 0.38 at TP4 and 0.22 at TP5) (Figure 2 and Supplementary Materials Table S1).

The initial CBC at baseline (TP1) was not significantly different between seropositive and seronegative patients (Supplementary Materials Table S2). Furthermore, no difference in immune response was observed by gender at TP3 and TP4 for all treatment groups, but significantly lower in male for AZ-AZ subgroups compared with other regimens (*p* = 0.041) (Supplementary Materials Table S3).

### 3.2. Subgroup Analysis between Low-Dose and High-Dose Radiation 

**Figure 3:**
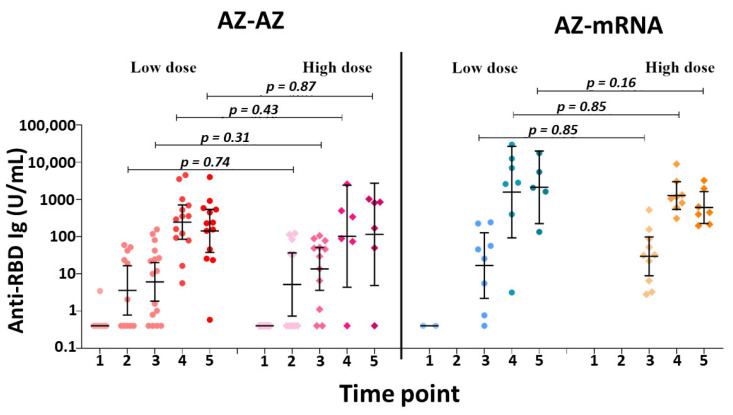
GMT and 95%CI for the anti-RBD total Ig levels at each time-point. comparing the treatment groups and the subgroups stratified by radiation dose. Anti-RBD Ig = immunoglobulins specific to the receptor-binding domain of the SARS-CoV-2 spike protein; AZ= AstraZeneca vaccine.

When stratified by radiation dose, there was no significant difference in GMT titers between the low-dose and high-dose groups (Figure 3 and Supplementary Materials Table S4). We further analyzed the complete blood count between the low-dose and high-dose groups and found no significant difference in hemoglobin, white blood cells, neutrophils, or platelets, however, there was a trend of higher lymphocytes in the high-dose group at TP1 (1860 cells/mm^3^ vs. 990 cells/mm^3^, *p* = 0.06) (Supplementary Materials Table S5).

### 3.3. Neutralization against SARS-CoV-2 Delta Strain and Omicron (BA.1) Strain

**Figure 4:**
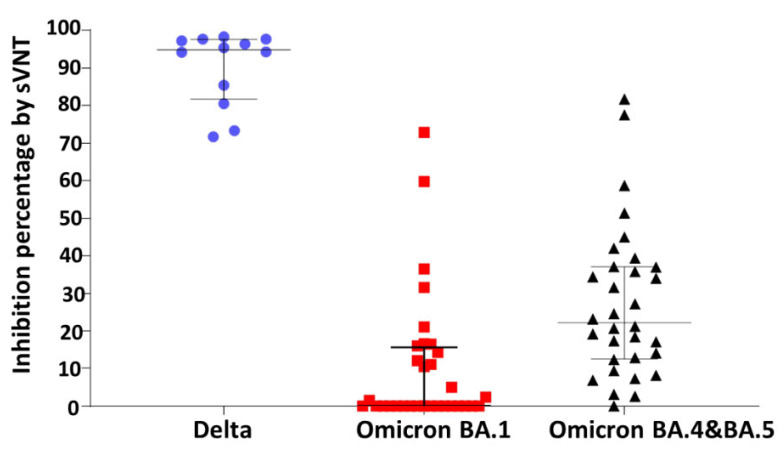
Percentage of surrogate virus neutralization test (sVNT) neutralization against the Delta, Omicron BA.1 and BA.4&BA.5 strains. The blue dot, red square, and black triangle represent the inhibition percentage by sVNT for the Delta, Omicron BA.1 and BA.4&BA.5 strains, respectively.

The neutralizing activity against the SARS-CoV-2 variants was determined using sVNT in a subset of 12 samples for the Delta strain and 32 samples for the Omicron strains at TP4. The results were consistent with the detection rates of neutralization, defined as inhibition ≥ 30%, which was 100% (95% CI, 73.5–100%), 12.5% (95% CI, 3.5–29%), and 40.6% (95% CI, 23.7–59.4%) against the Delta, Omicron BA.1 and BA.4&5 strain, respectively. Correspondingly, the median percentage of sVNT neutralization was 95% (IQR, 83–97%), 0% (IQR, 0–15.2%), and 22.2% (IQR, 12.7–37.1%) against the Delta, Omicron BA.1, and BA.4&5 strain, respectively (Figure 4).

### 3.4. Adverse Effects after Vaccination

**Figure 5:**
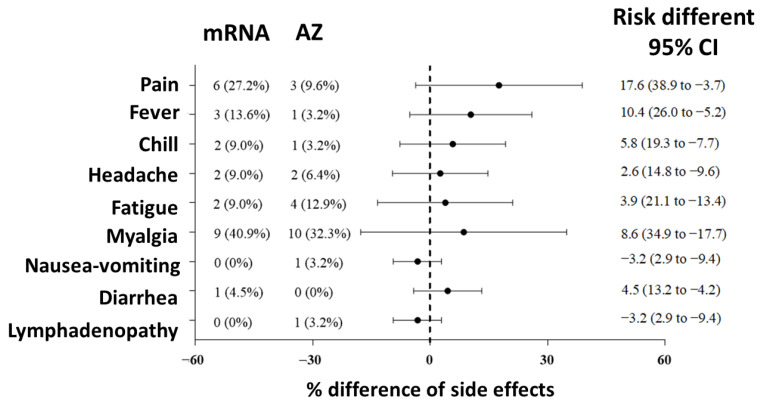
Comparison of the adverse effects of the AZ vaccine and mRNA vaccine after the second dose vaccination.

Most of the patients experienced mild adverse effects from the COVID-19 vaccine after the first and second dose. The patients either self-recovered or received conservative treatment. The most common side effects reported within seven days after vaccination were myalgia and pain. After the first and second dose, mild myalgia occurred in 19 patients (35.8%) for each dose. Pain at site of injection occurred in 19 patients after the first dose which was mild in 18 patients (33.9%) and severe in 1 patient (1.9%). After the second dose, 9 patients (17%) reported pain which was mild in 3 patients (9.6%) receiving AZ and 5 patients (22.7%) receiving mRNA. One patient had severe pain after the second dose of mRNA vaccine (Supplementary Materials Table S6). Compared with the AZ group, adverse effects occurred more frequently in the mRNA group after the second dose of vaccination (Figure 5). No radiation recall phenomenon was observed.

## 4. Discussion

To the best of our knowledge, this is the first report of the immunogenicity and safety profile of the COVID-19 vaccines in patients undergoing radiotherapy at the time of vaccination. Our study found fair immunogenicity following a single-dose vaccination (seropositivity rate 52.4%) that improved after the second dose (seropositivity rate 95–100%) with a durable humeral response for at least three months after two vaccinations (seropositivity rate 89.5–100%). The improvement in seroconversion after the second dose was comparable with other studies (Table 3). 

According to the NCCN guidelines, the recommended timing of COVID-19 vaccination for cancer patients who are on active cancer treatment is after recovery from nadir of myelosuppression [3]. However, cancer patients who are on active treatment are more vulnerable to COVID-19 infection and should be the top-priority for the vaccination. Meanwhile, anticancer treatments have negative effects on the proliferation of cancer cells and healthy rapidly dividing cells in the bone marrow. Several studies demonstrated that patients with solid tumors undergoing active treatment had a lower immune response in terms of seroconversion and antibody concentration. The seropositivity rate after the second dose was 52–85% in the cancer patients who were undergoing active treatment, 65–91% in the cancer patients who were not receiving treatment, and 98–100% in the healthy controls [4,11,15]. The largest study in Thailand reported an immunogenicity of 61% and 94% after the first and second dose of AZ, respectively [10]. 

Radiotherapy is one of the mainstay cancer treatments and may suppress the immune response as well as marrow function, resulting in less effective immunization by COVID-19 vaccines [12]. Nevertheless, data on the immunogenicity and safety profile in solid tumor patients who were receiving radiotherapy is scarce and there is a variety of national policy of vaccination program. A British study in 8517 participants who received AZ and mRNA vaccines reported a seropositivity rate of 55% after the first dose and approached 100% after the second dose with a limited number of radiotherapy patients (N = 5) [9]. Our study recruited 53 radiotherapy patients and the results were consistent with previous studies that the immunologic response after the first vaccination dose was inadequate (52.4%) in solid tumor patients who were on active cancer treatment, regardless of initial CBC. Therefore, strategies to enhance immune function might be beneficial, for example, the early administration of second dose and pre-exposure prophylaxis with monoclonal antibodies. Our study found that the heterologous AZ-mRNA schedule (administered with a 4- to 8-week interval) could be safely administered and induced relatively higher anti-RBD Ig levels compared with the AZ-AZ regimen that was administered 10–12 weeks apart. The studies in a healthy population receiving the heterologous AZ-mRNA combination regimen also demonstrated a potent induction of antibody response and neutralization against the SARS-CoV-2 Delta variant [16,17,18]. Therefore, the heterologous regimen could potentially enhance the protection from COVID-19 infection. 

Compared with healthy individuals, several studies reported lower and delayed immunogenicity after COVID-19 vaccination in cancer patients, especially those with a hematologic malignancy [8,9,19]. This was consistent with our results that the GMT level was lower in cancer patients compared with healthy individuals. Pre-exposure prophylaxis with monoclonal antibodies, AZD7442 (toxagevimab co-packaged with cilgavimab; AstraZeneca, Cambridge, UK; Evusheld^®^), has been authorized by the US Food and Drug Administration (FDA) under an Emergency Use Authorization (EUA). It is an effective option for cancer patients who have received immunosuppressive treatments and may not mount an adequate immune response to COVID-19 vaccination [15,20,21]. Furthermore, other preventive strategies for COVID-19 infection are of importance, including the universal precaution campaign of using a face mask, frequent hand washing, and social distancing.

We hypothesized that the patients in the low-dose group received palliative treatment due to a worse disease prognosis or poor clinical performance, thus they likely had impaired immunologic function. However, there was no significant difference in the seropositivity rate and antibody concentration between the groups stratified by radiation dose, high-dose (>50 Gy) or low-dose (≤50 Gy). It is worth noting that the sample size was too small to elaborate on its impact. 

The safety profiles of our patients indicated that the COVID-19 vaccinations were well-tolerated and were similar to previous studies using AZ or mRNA vaccines [10,22,23,24]. The local and systemic adverse events reported were mostly mild or moderate and were less frequent for the AZ vaccine than the mRNA vaccine. No radiation recall phenomenon was reported. The overall incidence of adverse reactions was slightly higher in the healthy population, which might be due to a better immune reaction [14,23,25]. 

Studies reported that the acceptance rate of COVID-19 vaccination among cancer patients was lower than the normal population, ranging from 18–62% [26,27,28]. The reasons for rejection or hesitancy among the public were doubting the vaccine’s efficacy and its side effects. Cancer patients were also concerned about the effect of the vaccine on their current anti-cancer therapy. Therefore, the results of this study provide data to support the feasible and safe administration of vaccines in cancer patients undergoing treatment.

The limitations of this study included the small sample size and limited availability data of the healthy cohort and missing data of the cancer cohort, thus meticulous analysis and comparison were hampered and the interpretation of the results should be made with caution. However, our study reflected the real-world population in view of the switching vaccination program in Thailand. Another limitation was that we assessed the immunogenicity using the anti-RBD Ig level which is a surrogate outcome for the vaccine’s efficacy, not live virus neutralization. Hence, surrogate immunological outcomes were employed in this study since the surrogate neutralization titers showed a good correlation with the antibody level and a low rate of natural infection was detected [29].

## 5. Conclusions

The COVID-19 vaccines can be safely administered to cancer patients undergoing radiation treatment and the second dose is mandatory to enhance immunogenicity approaching that of the healthy population with a durable response. Our results demonstrated that the effects of the radiotherapy dose might be non-existent on the immunologic response after vaccination. Additional studies should investigate additional doses of the vaccine and the effects of radiotherapy volume on immunologic response.

## Figures and Tables

**Table 1:** Baseline patient characteristics.

		N (%)
Sex	Male	19 (35.8)
	Female	34 (64.2)
Age	Mean ± SD (years)	56 ± 13.6
Primary Tumor	CNS	5 (9.4)
	Head and neck	17 (32.1)
	Thorax (breast, lung)	19 (35.8)
	Abdomen	11 (20.7)
	Bone	1 (1.9)
Stage	I-II	13 (24.5)
	III-IV	40 (75.5)
Systemic treatment	Chemotherapy	24 (45.3)
Steroids	Yes	9 (17)
Smoking	Current smoker	7 (13.2)
	Previous smoker	4 (7.5)
	Never smoke	42 (79.2)

SD = standard deviation; CNS = central nervous system.

**Table 2:** Radiation treatment.

	N (%)
Aim of treatment	
-Curative aim	39 (73.6)
-Palliative aim	14 (26.4)
Number of fraction (fractionation) (median, IQR)	25 (10–30)
Dose in Gy (median, IQR)	45 (40–63)
Technique	
-3D-CRT	17 (32.1)
-IMRT/VMAT	31 (58.5)
-SRS/SRT/SBRT	4 (7.5)
-Proton therapy	1 (1.9)

IQR = interquartile range; 3D-CRT = three-dimensional conformal radiotherapy, IMRT = intensity modulated radiotherapy; VMAT = volumetric arc modulated radiotherapy; SRS = stereotactic radiosurgery; SRT = stereotactic radiotherapy; SBRT = stereotactic body radiotherapy.

**Table 3:** Comparison of the seroconversion and antibody response after vaccination in the cancer patients undergoing active treatment.

Studies	Vaccine	Number of Cancer Patients (Active Treatment/All)	Seropositivity		Antibody Titers at 4 Weeks Post-Vaccination	
			After the First Dose	After the Second Dose	After the First Dose	After the Second Dose
Teeyapan	AZ-AZ	385/385	60.8%	93.6%	3.4 U/mL	224.5 U/mL
Nelli	PZ-PZ	285/366	52%	91.2%	62 AU/mL	1530 AU/mL
Cavanna	PZ-PZ	219/257	NA	76%	NA	118 AU/mL
Yasin	PZ-PZ	426/776 309 CMT 117 Targeted/IO	NA	85.2% 78.6% 84.6%	NA	NA
Bowes	PZ-PZ MDN-MDN JJ	*/33	NA	NA	NA	263 U/mL
Ours	AZ-AZ AZ-PZ	53	54.2%	95% 100%	4.3 U/mL	188.4 U/Ml 1400.8 U/mL

AZ = AstraZeneca; PZ = Pfizer; MDN = Moderna; JJ = Johnson & Johnson; NA = not available; * not known number of patients on active treatment.

## Data Availability

The research data are stored in an institutional repository and will be shared upon request to the corresponding author.

## Supplementary Materials

The following supporting information can be downloaded at: https://www.mdpi.com/article/10.3390/vaccines11071135/s1, Figure S1: A flow diagram of data collection at each time-point; Table S1: The GMT and GMR for the anti-RBD total Ig levels of the treatment groups; Table S2: Comparison of the baseline (time-point 1) complete blood count analysis according to seroconversion status at 1 month after first vaccination (time-point 2); Table S3: Seropositivity rate of the treatment groups stratified by gender; Table S4: The GMT and GMR for anti-RBD total Ig levels of the low-dose and high-dose subgroups; Table S5: Comparison of the complete blood count analysis in the low-dose and high-dose groups at time-point 1 and 3; Table S6. Adverse effects of the COVID-19 vaccine.
