# Supplementary material for: Safety and Immunogenicity of Homologous and Heterologous Adenoviral-Vectored and mRNA COVID-19 Vaccine Regimens in Radiotherapy Patients
Source: Vaccines (Basel). 2023 Jun 23;11(7):1135. doi: 10.3390/vaccines11071135 (PMC10383644; doi:10.3390/vaccines11071135)
Supplement: Supplementary file 1 [file vaccines-11-01135-s001.zip › vaccines-2288079-supplementary.pdf]

Supplementary Materials

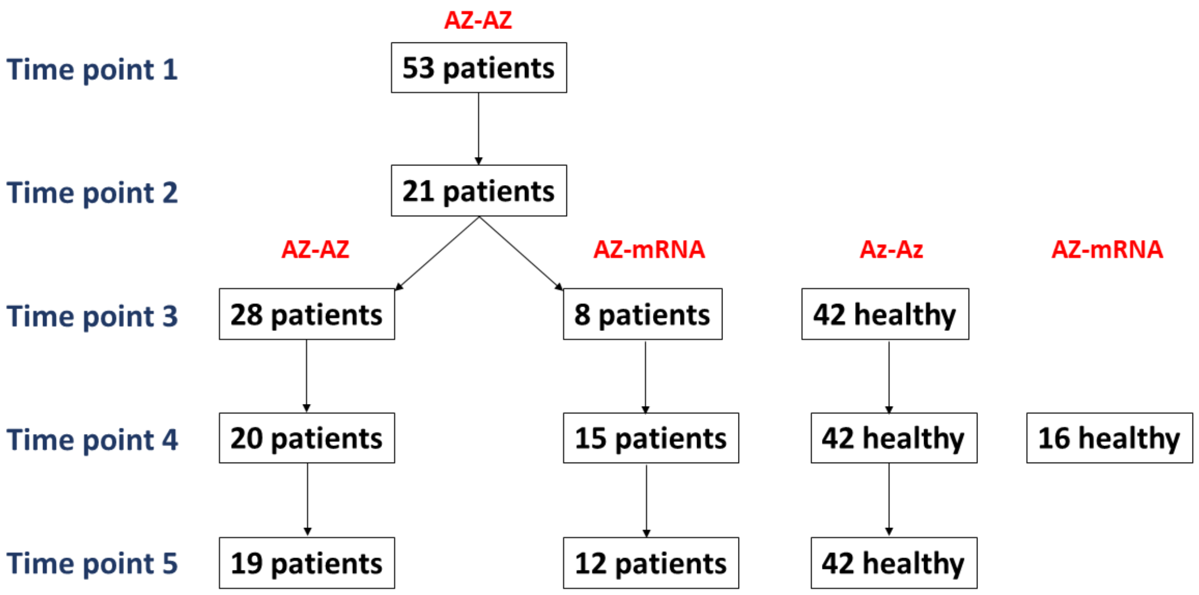

Figure S1. A flow diagram of data collection at each time-point.

Table S1. The GMT and GMR for the anti-RBD total Ig levels of the treatment groups.

	AZ-AZ group	AZ-mRNA group	Healthy controls (AZ-AZ)	Healthy controls (AZ-mRNA)
**Time-point 3**				
GMT (95% CI)	9.3 (3.9-22.5)	18.4 (6.5-52.2)	76 (55.3-105)	NA
GMR (95% CI)<br>P-value	0.12 (0.05-0.28)<br><0.001	0.24 (0.1-0.61)<br>0.003	1<br>Ref	NA
**Time-point 4**				
GMT (95% CI)	188.4<br>(67.1-529)	1400.8<br>(429.5-4566)	945<br>(708-1261)	5169.9<br>(3582.2-7461.5)
GMR (95% CI)<br>P-value	0.04 (0.01-0.10)<br><0.001	0.27 (0.09-0.79)<br>0.02	0.18 (0.08-0.44)<br><0.001	1<br>Ref
**Time-point 5**				
GMT (95% CI)	133.1 (41.9-423)	1018.8(395-2628)	538 (389-744)	NA
GMR (95% CI)<br>P-value	0.25 (0.1-0.59)<br>0.002	1.89 (0.68-5.28)<br>0.22	1<br>Ref	NA

GMT=geometric mean titer; GMR=geometric mean titer ratio; CI=confidence interval; Ref=reference; NA=not applicable.

Table S2. Comparison of the baseline (time-point 1) complete blood count analysis according to seroconversion status at 1 month after first vaccination (time-point 2).

	Seropositivity	Seronegativity	P-value
**Hemoglobin,** mean(SD)	12.1 (1.5)	11.1 (0.9)	0.09*
**Platelets,** mean(SD)	266,500 (62,100)	292,800 (90,100)	0.45*
**White blood cell**, median(IQR)	5,345 (4,660-6,900)	5,260 (4,380-8,760)	0.89¥
**Neutrophil**, median(IQR)	3,690 (2,610-4,330)	3,830 (2,770-5,250)	0.78¥
**Lymphocyte**, median(IQR)	1,015 (940-1,900)	1,510 (780-2,140)	0.94¥

* using two sample independent t-test; ¥ using Wilcoxon rank-sum test.

Table S3. Seropositivity rate of the treatment groups stratified by gender.

	AZ-AZ group	AZ-mRNA group	Healthy controls (AZ-AZ)	P-value
**Time-point 3**				
Male	12/14 (85.7)		21/21 (100)	0.07
Female	21/26 (80.8)		21/21 (100)	0.08
P-value	0.72	0.50	-	
**Time-point 4**				
Male	6/7 (85.7)	4/4 (100)	21/21 (100)	0.34
Female	13/13 (100)	11/11 (100)	21/21 (100)	-
P-value	0.35	-	-	
**Time-point 5**				
Male	4/6 (66.7)	3/3 (100)	21/21 (100)	0.041
Female	13/13 (100)	9/9 (100)	21/21 (100)	-
P-value	0.09	-	-	

Table S4. The GMT and GMR for anti-RBD total Ig levels of the low-dose and high-dose subgroups.

	All	Low-dose	High-dose	GMR (95%CI)	p-value
**AZ-AZ group**					
TP1	<0.4	<0.4	<0.4	-	-
TP2	4.3 (1.4-13)	3.6 (0.8-16.5)	5.2 (0.7-36.5)	0.69 (0.07-6.82)	0.74
TP3	9.3 (3.9-22.5)	6.4 (1.7-23.1)	15.7 (4.2-59.4)	0.41 (0.07-2.43)	0.31
TP4	188.4 (67.1-529)	244.9 (84.6-708.8)	102.2 (4.3-2402.3)	2.4 (0.24-23.49)	0.43
TP5	133.1 (41.9-423)	142 (37.7-536.3)	115.4 (4.9-2734)	1.23 (0.09-16.07)	0.87
**AZ-mRNA group**					
TP1	<0.4	<0.4	<0.4	-	-
TP2	NA	NA	NA	-	-
TP3	18.4 (6.5-52.2)	16.7 (2.2-127.2)	20.3 (5.8-71.8)	0.82 (0.09-7.17)	0.85
TP4	1400.8 (429.5-4566)	1571.5 (92.7-26634)	1266.6 (536.3-2991.4)	1.24 (0.1-14.71)	0.85
TP5	1018.8(395-2628)	2116 (224-19984)	604 (226-1618)	3.5 (0.56-22.04)	0.16

Data presents geometric mean titer (GMT) with 95% confidence interval (CI), High-dose group is used as reference. *GMR= Geometric mean titer ratio; TP=Time point; NA=not available*

Table S5. Comparison of the complete blood count analysis in the low-dose and high-dose groups at time-point 1 and 3.

	All	Low dose	High dose	P-value
**Hemoglobin**				
TP1	11.6 (10.8-12.3)	11.6 (11-12.3)	11.5 (10.1-12.9)	0.83
TP3	11.2 (9.9-12.1)	10.7 (8.9-11.9)	11.4 (10.5-12.2)	0.17
**Platelets**				
TP1	284 (244-320)	251 (240-297)	304 (246-377)	0.09
TP3	247 (223-291)	231 (191-292)	253.5 (224-286)	0.45
**White blood cell**				
TP1	5570 (4660-7760)	5245 (4370-6600)	6920 (5160-8760)	0.11
TP3	5620 (4640-7330)	5620 (4630-7330)	5750 (4640-8330)	0.83
**Neutrophil**				
TP1	3900 (2860-5250)	3115 (2770-4610)	4330 (3410-5550)	0.27
TP3	3910 (3100-5590)	3850 (2980-5590)	4085 (3100-5740)	0.86
**Lymphocyte**				
TP1	1150 (940-1930)	990 (870-1790)	1860 (1010-2510)	0.06
TP3	1210 (750-1830)	980 (650-1660)	1265 (860-1870)	0.45

TP=Time point.

Table S6. Adverse effects of the COVID-19 vaccine.

	Dose 1 (N=53) N (%)	Dose 2, N (%)		
		AZ (N=31)	mRNA (N=22)	Total (N=53)
**Pain (mild)**	18 (33.9)	3 (9.6)	5 (22.7)	8 (15.1)
Pain (severe)	1 (1.9)	0 (0)	1 (4.5)	1 (1.9)
Fever (mild)	10 (18.9)	1 (3.2)	3 (13.6)	4 (7.5)
Chill (mild)	2 (3.75)	0 (0)	1 (4.5)	1 (1.9)
Chill (severe)	2 (3.75)	1 (3.2)	1 (4.5)	2 (3.8)
Headache (mild)	6 (11.3)	2 (6.4)	2 (9)	4 (7.5)
Fatigue (mild)	5 (9.4)	4 (12.9)	1 (4.5)	5 (9.4)
Fatigue (moderate)	2 (3.8)	0 (0)	1 (4.5)	1 (1.9)
Myalgia (mild)	19 (35.8)	10 (32.3)	9 (40.9)	19 (35.8)
Nausea-vomiting (mild)	2 (3.8)	1 (3.2)	0 (0)	1 (1.9)
Diarrhea (mild)	1 (1.9)	0 (0)	1 (4.5)	1 (1.9)
Lymphadenopathy (mild)	1 (1.9)	1 (3.2)	0 (0)	1 (1.9)
Swelling	2 (3.8)	0 (0)	0 (0)	0 (0)
